# Expanding and Evaluating Public Satisfaction with Wildlife Governance: Insights from Deer Management in Indiana, USA

**DOI:** 10.1007/s00267-022-01698-5

**Published:** 2022-08-23

**Authors:** Taylor R. Stinchcomb, Zhao Ma, Robert K. Swihart, Joe N. Caudell

**Affiliations:** 1 Department of Forestry & Natural Resources, Purdue University, West Lafayette, IN USA; 2 Indiana Department of Natural Resources Division of Fish & Wildlife, Bloomington, IN USA

**Keywords:** Governance, Public trust, Satisfaction, Social science, White-tailed deer, Wildlife management

## Abstract

Wildlife agencies in North America desire to incorporate broader public interests into decision-making so they can realize the principle of governing wildlife in the public trust. Public satisfaction is a key component of good governance but evaluating satisfaction with wildlife management focuses on traditional user experiences rather than perceptions of agency performance. We draw from political science, business, and conservation social science to develop a multidimensional concept of satisfaction with wildlife management that includes agency performance, service quality, trust in the managing agency, and informational trust. We use data collected from a 2021 survey of Indiana residents to analyze the social and cognitive determinants of satisfaction with white-tailed deer (*Odocoileus virginianus*) management. Quantile regression models revealed that respondents’ acceptability of management methods and deer-related concerns most strongly affected performance and quality components, whereas respondent characteristics mostly affected trust components of the index. Future research should associate satisfaction with key variables we did not fully capture including perceived control, psychological distance, and norms of interaction between wildlife agencies and the public. Expanding agency conceptions of public satisfaction represents a critical step toward public trust thinking and the practice of good wildlife governance in North America.

## Introduction

Developing a more refined concept of public satisfaction with wildlife management could help agencies incorporate more diverse public interests into management planning and overcome inter-cultural differences with constituents (Association of Fish and Wildlife Agencies and The Wildlife Management Institute 2019). Close examination of public satisfaction enables agencies to determine why their constituents may be unsatisfied with certain management programs and identify with whom they should begin to improve their relationships. In the United States, under the Public Trust Doctrine, natural resource agencies hold and manage wildlife in trust for all its potential beneficiaries, i.e., all citizens (Jacobson et al. 2010). Consequently, improving relationships with individuals and groups and increasing public satisfaction with wildlife management is critical for agencies to realize this public trust model (Coleman et al. 2019).

In the North America, wildlife agencies have overwhelmingly focused on whether the traditional beneficiaries with a stake in wildlife management (i.e., hunters, anglers, recreators, or farmers) are satisfied with their wildlife interactions (Hendee 1974; Tian-Cole et al. 2002; Gruntorad et al. 2020). A few studies examine the satisfaction of these stakeholders with specific wildlife management methods (Schroeder et al. 2017; Pruitt et al. 2021), and acknowledge that such satisfaction is multidimensional (Hammitt et al. 1990; Vaske et al. 1986; Manfredo et al. 2004). These foci on consumptive users of wildlife or groups who are directly impacted by wildlife damage stem from a market-based model of public administration which conceptualizes citizens as customers of the wildlife agency. Unfortunately, public satisfaction has not been clearly defined in natural resources; satisfaction is often used interchangeably with the appeal, acceptability, or perceived effectiveness of certain management programs. Moreover, a customer-based approach does not adequately capture satisfaction with wildlife management because it does not incorporate broader public interests. Agencies must therefore work to identify diverse groups beyond the traditional stakeholders and clearly define satisfaction with wildlife management to truly serve the public trust (Jacobson et al. 2010).

### Service Quality and Agency Performance

Scholarship in business and governance provides insight into how natural resource agencies can define a multidimensional measure of public satisfaction. Market-based customer satisfaction depends critically on service quality and performance, and dissatisfaction occurs when individuals’ expectations exceed the quality or performance of a service they experience (Oliver 1980; Matzler et al. 2003; Tonge and Moore 2007). Park and protected area management uses multiple public preferences such as crowding, environmental conditions, and sensory experiences to measure the gap between visitors’ expected quality of services (e.g., public land activities, access, and maintenance) and visitors’ evaluation of their experience (Hollenhorst and Gardner 1994; Ryan and Cessford 2003; Tonge and Moore 2007). But another determinant of public satisfaction that agencies do not fully consider is how service quality relates to good governance. Good governance can be evaluated as both the quality of services provided by an agency and the integrity of democratic processes, as perceived by the public (Kelly and Swindell 2002; Van Ryzin 2007; Ariely 2013). Overall satisfaction with governance typically depends on the degree to which citizens perceive their governing bodies to be transparent and trustworthy (Park and Blenkinsopp 2011). To develop a more holistic measure of satisfaction, wildlife agencies thus need to consider how the public perceives their decision-making processes and governance capacities.

### Trust Between Agencies and Their Publics

Building trust between agencies and the public is a key practice of good governance, particularly in natural resource contexts (Stern and Coleman 2015). Like satisfaction, trust is based upon one’s expectations of the target’s intentions or behavior (Rousseau et al. 1998; Stern and Coleman 2015), and trust erodes when those expectations are unmet. When organizations betray workplace or public trust in their operations—through fraud, corruption, exploitation, or negligence—their employees retaliate through reduced effort or public protest, respectively (Gillespie and Dietz 2009). Similarly, distrust in the competencies or values of government agencies often motivates civic engagement as a means of dissent (Smith et al. 2013; Inglehart and Norris 2016). Recent events in Wisconsin, USA, provide a prime example of how trust in wildlife management agencies can quickly erode. During the wolf (*Canis lupus*) hunting season in February 2021, hunters killed 97 more wolves than the Wisconsin Department of Natural Resources had anticipated. This perceived oversight led to multiple lawsuits from animal rights groups and six Native American (Ojibwe) tribes, which resulted in a judicial injunction placed on the November wolf hunting season (Kaeding 2021; Richmond 2021). In this case, a lack of transparency about how the agency came to their initial quota and a lack of communication with non-hunting stakeholders fostered public dissatisfaction with the agency’s wolf management approaches and distrust in the agency’s competence and credibility (Stern and Coleman 2015; Manfredo et al. 2017). This transparency-trust relationship occurs in other wildlife management contexts (Sjölander-Lindqvist et al. 2015; Riley et al. 2018; Schmidt et al. 2018) and demonstrates a clear connection between informational and organizational trust (Denize and Young 2007). As such, evaluating public satisfaction in wildlife governance requires that agencies measure informational trust and transparency along with trust in their technical competencies and credibility.

Agency credibility is compromised when sectors of the public perceive management decisions to favor the interests of specific groups over others (Riley et al. 2018; Stinchcomb et al. 2022). The equitable inclusion and consideration of diverse public interests, known as procedural fairness, may be the most important predictor of trust between natural resource agencies and their constituents (Riley et al. 2018; Ford et al. 2020). The similarity that an individual perceives between his/her/their values and those of an agency also affects trust in its management decisions (Needham and Vaske 2008; Smith et al. 2013). But wildlife administrators often struggle to address imbalanced perceptions of procedural fairness and value congruence across constituents, likely because they lack awareness about the factors that constitute public trust in their agency. Such awareness is typically acquired by specialists in the human dimensions of wildlife management, but these individuals make up a tiny fraction of wildlife agency staff (Morales et al. 2021). Developing greater trust between agencies and their constituents is an important part of agencies becoming more relevant toa wider public. Recent guidance from the Association of Fish and Wildlife Agencies alluded to the factors that influence public trust in natural resource management, but it did not explicitly define trust nor its multiple dimensions (Association of Fish and Wildlife Agencies and The Wildlife Management Institute 2019). Therefore, agencies need to first understand the importance of procedural trust and its relation to satisfaction, then measure the determinants of trust among their constituents, and finally improve trust through visible efforts to make decision-making processes more inclusive, unbiased, and transparent.

### Individual Characteristics and Contexts

Trust between agencies and their constituents remains highly nuanced, context-bound, and dependent on characteristics of the agency or of interest and the local peoples, groups, or communities it serves (Saunders 2012). For example, Scandinavian communities place higher trust in government agencies than most European countries due to their collectivist cultures and extensive government provision of social benefits (Fitzgerald and Wolak 2016). In the North American Arctic, communities place lest trust in extractive industries or law enforcement and more trust in local fish and wildlife management agencies (Schmidt et al. 2018). Trust also varies with region, gender, and education level (Schmidt et al. 2018), and public satisfaction varies with individual characteristics, interpersonal history with the agency, and service inequalities (Kelly 2005). Among landowners in Texas, USA, and South Africa, satisfaction with private land conservation programs varied with place-based values or motivations and eroded when conservation programs did not meet their expectations for services like technical support or personal contact with program staff (Selinske et al. 2015). Analyzing individual, group, and community differences in expected and perceived agency performance is key to gauging public satisfaction with natural resource management. Further, these analyses can help agencies understand what, where, and why agency efforts and outcomes are inequitably distributed (Kelly 2005).

In review, prior research has revealed four major components that influence public satisfaction with natural resource governance: (i) performance of governing agencies; (ii) the quality of services they provide; (iii) trust between agencies and the public (referred to hereafter as “agency trust”); and (iv) individual characteristics and local contexts. Moving beyond a simple rating, satisfaction can be conceptualized as the gap between expectations and outcomes, where outcomes for the public include performance, service quality, trust, and information dissemination. Despite their conceptual linkages, studies in wildlife management have rarely brought perceptions of management performance, agency trust, and informational trust together to provide a holistic view of public satisfaction with wildlife agencies. Here, we combine these constructs into one index to tease apart the various determinants of satisfaction with the management of white-tailed deer (*Odocoileus virginianus*, hereafter “deer”) in Indiana, USA. Our index, constructed with survey data, provides a tool for agencies to identify which segments of the public are dissatisfied with wildlife management and what drives that dissatisfaction. Applying such an index will help agencies incorporate broader public interests into wildlife management, thus moving closer to the public trust ideal.

### Research Context and Objectives

Since their reintroduction to Indiana in 1934, deer populations have proliferated widely across the state (Brown and Parker 1997). Deer impact forest ecosystems, agricultural activities, and public health and safety (Brown and Parker 1997; Curtis and Lynch 2001; Marcoux and Riley 2010; INDFW Indiana Department of Fish & Wildlife 2011; Urbanek et al. 2015). Yet people also derive benefits from deer including hunting opportunities, awe, enjoyment, and cultural connection (King 2002; Hicks 2017; McIntosh and Wright 2017).

Deer management in Indiana falls under the authority of the Indiana Department of Natural Resources (IN-DNR) Division of Fish & Wildlife (DFW). As part of their annual deer management survey, the IN-DNR asks licensed hunters and anglers to rate their satisfaction with deer management on a scale 1 to 100. In 2022, the survey was expanded to subscribers of the DFW’s Wild Bulletin newsletter and included questions about public trust in the IN-DNR’s competency. But the survey neither includes other dimensions of satisfaction nor reaches Indiana residents for whom the DFW has no contact information. Thus, the IN-DNR sought a more inclusive and multidimensional measure of public satisfaction with deer management.

**Fig. 1:**
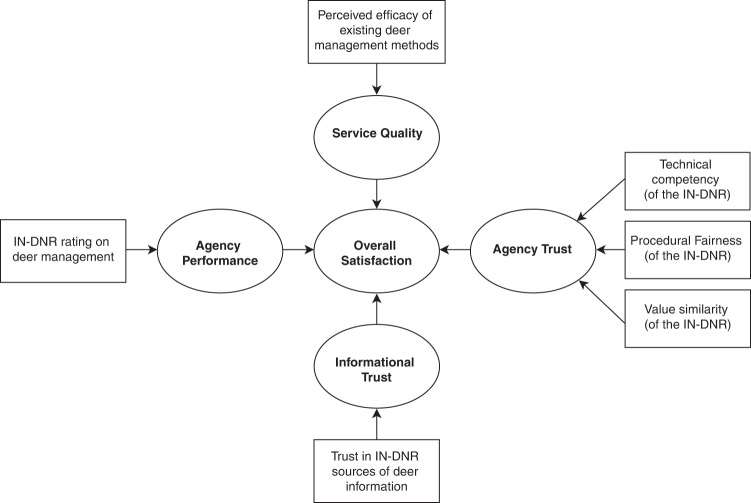
Hypothesized composition of satisfaction with deer management in Indiana. Ovals represent latent constructs derived from survey items. Rectangles represent items measured directly in the survey. IN-DNR = Indiana Department of Natural Resources

Based on the literature, we presume satisfaction with deer management to be comprised of the following four components: an individual’s (1) perception of the IN-DNR’s performance in deer management, (2) perception of the efficacy of existing management approaches, (3) trust in the IN-DNR’s technical capacity, procedural fairness, and value similarity, and (4) trust in information coming from the IN-DNR (Fig. 1). Scholars have established a clear relationship among stakeholder beliefs, attitudes, and acceptance of wildlife management actions (Loker et al. 1999; Fulton et al. 2004; Whittaker et al. 2006; Bruskotter et al. 2009), which likely influences perceptions of agency performance and management efficacy (Schroeder et al. 2021a). Values for and attitudes toward wildlife also relate closely to agency and informational trust (Manfredo et al. 2017; Schroeder et al. 2021b). Finally, trust in agencies varies with individual characteristics (Saunders 2012; Schmidt et al. 2018). We therefore expect multiple factors to predict satisfaction, including general attitudes toward deer, concerns about deer populations, beliefs about hunting, wildlife value orientations, respondent self-identity, and other individual characteristics.

Our study has three main objectives. First, we develop a satisfaction index from survey questions on the IN-DNR’s performance, the quality of deer management, agency trust, and informational trust (Fig. 1). Second, we determine which major psychological and demographic characteristics most influence respondents’ satisfaction scores and how these predictors vary with index components. Finally, we evaluate the utility of our index for wildlife and natural resource management.

## Methods

### Data Collection

To quantify Indiana residents’ perceptions of deer management and the IN-DNR, we implemented a statewide survey from June to August 2021. Prior to data collection, our study design was approved by the Institutional Review Board of Purdue University, protocol number 1902021653. We sampled 6000 residents randomly within a 2 × 4 stratified design. The higher-order stratum separated residents equally into 3000 license-holders with the Indiana DFW and 3000 non-license-holders (non-DFW). Within each of these strata, we randomly sampled 750 tax parcel addresses from four landscape types: forest, farmland, developed area, and “integration.” We drew the “integration” sub-sample from addresses within 6.4 × 6.4 km (4 × 4 mi) grids used in ongoing studies by colleagues to collect ecological data. We subsampled by landscape to obtain a wide range of possible human-deer interactions among respondents, and thus obtain a diversity of beliefs, attitudes, and individual experiences that we expect to affect respondent satisfaction with deer management. We used ESRI ArcPro software to sample each stratum. Data were obtained from the IndianaMap geospatial database, the Indiana Department of Local Government and Finance, and the Indiana DFW. Addresses were checked for duplicates, blanks, and public or corporate ownership and re-sampled as necessary.

Survey dissemination followed Dillman et al.’s (2014) Tailored-Design Method. We sent all questionnaires via postal mail with an option to take the survey online indicated on a cover letter. Each sampled resident was sent a pre-notification postcard, followed a week later by a survey packet, and a reminder postcard sent to non-respondents 2 weeks after first contact. We conducted three mailings in total, with the final two mailings sent to residents who had not yet responded via mail or online.

We checked for non-response bias by comparing key demographic characteristics of our sample to the corresponding census data for Indiana. We used chi-squared tests to determine whether significant differences existed between our sample proportions and those expected at the state level. We consider the implications of non-response bias in our Discussion.

### Data Analysis

To create an index of satisfaction, we combined the following items from our survey to measure the four components listed above: overall rating of the IN-DNR’s deer management, effectiveness ratings for existing deer management approaches, level of trust in the IN-DNR, and level of trust in information sources from the IN-DNR (fig. 1). We assessed the inter-item reliability for each component using Cronbach’s alpha (Cronbach 1951). Exploratory factor analyses were conducted to determine whether survey responses used to measure each component of the satisfaction index loaded onto the expected number of factors. For cases in which all items sorted onto a single factor with similar loadings (±0.05), we averaged responses across the survey questions. For cases that loaded onto a single factor but with variable factor loadings (±0.15), we multiplied each item by its factor loading then summed the items together to create a composite variable. We generated the final satisfaction index by summing each respondent’s rating of the IN-DNR with their composite scores for management efficacy, agency trust, and informational trust.

Next, we used the satisfaction index as the dependent variable in multiple regression analyses to determine what variables best predict satisfaction. Independent variables in the full additive model included a respondent’s average acceptability of lethal management methods (e.g., licensed hunting, culling, Community Hunting Access Programs (CHAPs)) and of nonlethal management methods (e.g., contraception, translocation, information provision), average level of deer-related direct concerns (e.g., forest damage, crop damage, vehicle collisions) and indirect concerns (e.g., disease transmission, hunting opportunities, population sizes, urban area management), average general attitude toward deer (positive/negative), average agreement with beliefs about deer hunting, and respondent characteristics including self-identity, hunter status, wildlife value orientation, whether they allow hunting on their land, organizational membership, political ideology, gender, and having graduated college. Lethal management, nonlethal management, direct concerns, and indirect concerns were all composite variables identified in an exploratory principal component factor analysis. We did not identify additional factors within these groupings.

We used quantile regression with bootstrapped standard errors to accommodate heteroscedasticity, kurtosis, and outliers in our independent variables. Quantile regression is robust to heteroscedastic errors, outliers, and spiked survey responses (Cade and Noon 2003; Sauzet et al. 2019). Instead of estimating the mean of the dependent variable and minimizing the sum of squared residuals, our quantile regression models estimated the median (50^th^ quantile) conditional on predictor variables and minimized the sum of the absolute residuals (Koenker and Bassett 1978; Cade and Noon 2003).

To examine the patterns of variation within our final satisfaction index, we conducted quantile regressions using each of the index components as dependent variables. This allowed us to compare the influence of independent variables across each component of the index. Each regression equation was the same as that for the overall satisfaction index, but with management efficacy, IN-DNR score, agency trust, and informational trust each substituted in place of the dependent variable. All analyses were conducted with the *quantreg* package in STATA 16.

## Results

### Descriptive Statistics

We received 1806 survey responses with 500 undeliverable, deceased, or otherwise ineligible for a response rate of 33%. Survey respondents were 76% male and 23% female, overwhelmingly White/Caucasian (92%), with an average age of 60 and an average of 51 years of residency in Indiana (Table 1). Respondents self-identified primarily as either rural residents (43%) or urban residents (25%), with 12% identifying as primarily deer hunters and 12% as primarily farmers or ranchers (Table 1). When asked to select multiple options with which they self-identify, 37% of respondents self-identified as a deer hunter, even though it was not necessarily their primary identity (hereafter referred to as “hunters”). Respondents were also asked whether they allow licensed hunting on their property. Respondents split equally between Yes (36%) and No (36%), with the remaining 28% indicating that either local ordinances prohibit hunting, or they don’t own private land suitable for hunting (Table 1). We grouped these respondents into a “cannot allow hunting” category and refer to them as such throughout this paper. Most respondents held pluralist wildlife value orientations (42%), followed closely by traditionalist orientations (35%). We summarize the distribution of responses to variables included in quantile regression models in Online Resource 1.

**Table 1:** Observed proportions on characteristics of survey respondents (*n* = 1806) and overall proportions from the Indiana population

Variable	*n*	Sample proportion	Statewide proportion^a^
Primary Self-Identity	1771		
Farmer/Rancher		0.12	0.01^b^
Woodland Owner		0.08	0.04^c^
Deer Hunter		0.12	0.04^d^
Urban Area Resident		0.25	0.62^e^
Rural Resident		0.43	0.38^e^
Identifies as a deer hunter	1806	0.37	0.04^d^
Allows Hunting on Property	1761		
Yes		0.36	n/a
No		0.36	n/a
Cannot (“local ordinances prohibit hunting” + “I don’t own private land suitable for hunting”)		0.28	n/a
Gender	1749		
Man		0.76	0.49
Woman		0.23	0.51
Ethnicity	1717		
White/Caucasian		0.92	0.62
Black/African American		0.01	0.12
Hispanic/LatinX		0.00	0.19
Asian/Asian American		0.01	0.06
Native American/Alaska Native		0.00	0.01
Pacific Islander		0.00	0.00
Household Income	1562		
<$50,000		0.25	0.43
$50,000–$99,999		0.38	0.32
$100,000–$199,999		0.30	0.20
>$200,000		0.07	0.05
Highest Education	1731		
school or less		0.32	0.44
Associates degree or some college		0.31	0.29
College or graduate degree		0.37	0.27
Age	1806		
18–24		0.01	0.10
25–44		0.15	0.26
45–64		0.39	0.25
65 and older		0.41	0.17

^a^Data from 2019 U.S. Census and American Community Survey, unless otherwise noted. Total Population of Indiana = 6.732 million

^b^2017 USDA Census of Agriculture, Indiana

^c^2018 USDA National Woodland Owner Survey

^d^2019 Indiana White-Tailed Deer Report

^e^Purdue University Extension Report, “Population Trends in Indiana.” Proportions of the population living in urban counties and mixed rural or rural counties

### Predictors of Satisfaction

Significant predictors of satisfaction included management acceptability, deer-related concerns, general deer attitudes, beliefs about hunting, and respondents’ characteristics which include self-identity, wildlife value orientation, and whether they allowed hunting on their property.

Respondents’ ratings of the acceptability of deer management methods, both lethal and nonlethal, significantly affected median satisfaction with deer management. A standard deviation increase in acceptability of lethal management methods (+3.4 units) was associated with a 1.02 unit increase in median predicted satisfaction (slope = *β* = 0.30, *p* < 0.001, Table 2). A standard deviation increase in acceptability of nonlethal management methods (+2.8 units) corresponded to a 0.41 unit increase in median predicted satisfaction with management (*β* = 0.15, *p* = 0.036, Table 2).

**Table 2:** Quantile regression slope coefficients (*β*) for predictors of overall satisfaction with deer management. Base category for categorical variables in parentheses

	Predicted median slope with satisfaction		
Variable	*β*	se	*p*
Management acceptability			
Lethal	0.30	0.06	<0.001
Nonlethal	0.15	0.07	0.036
Deer Concerns			
Direct	0.03	0.07	0.715
Indirect	−0.01	0.05	0.901
Deer Attitudes	0.62	0.21	0.003
Hunting Beliefs	0.49	0.06	<0.001
Political Ideology	−0.29	0.13	0.024
Primary Self-Identity (Rural Resident)			
Farmer/Rancher	−1.09	0.64	0.088
Woodland Owner	−0.37	0.06	0.535
Deer Hunter	−0.65	0.69	0.342
Urban Resident	−0.95	0.54	0.078
Wildlife Value Orientation (traditionalist)			
Mutualist	0.25	0.59	0.669
Pluralist	1.17	0.37	0.002
Distanced	−1.48	1.29	0.249
Organizational Membership (None)			
Hunting	0.60	0.88	0.495
Environmental	0.99	0.72	0.169
Animal Welfare	0.77	0.61	0.204
Allows Hunting (Yes)			
No	0.64	0.40	0.111
Cannot	1.29	0.50	0.009
College Graduate (vs. No 4-yr Degree)	−0.05	0.39	0.900
Deer Hunter (vs. Not a Deer Hunter)	−0.08	0.45	0.866
Woman (vs. Man)	−0.85	0.45	0.060
Constant	6.94	0.59	<0.001

Levels of concern about both direct and indirect impacts from deer did not affect median satisfaction with deer management (Table 2). In contrast, respondents’ attitudes toward deer and beliefs about hunting showed strong effects on median satisfaction with deer management (Table 2). A standard deviation change in attitudes toward deer (0.96 units more positive) resulted in a 0.59 unit increase in median predicted satisfaction with management (*β* = 0.62, *p* = 0.003). With a standard deviation change in hunting beliefs (3.36 units more positive), median predicted satisfaction increased by 1.63 units (*β* = 0.49, *p* < 0.001, Table 3).

**Table 3:** Quantile regression slope coefficients for DNR performance and management efficacy components of satisfaction. Base category for categorical variables in parentheses

	DNR Score			Management Efficacy			Agency Trust			Informational Trust		
Variable	*b*	se	*p*	*b*	se	*p*	*b*	se	*p*	*b*	se	*p*
Management Acceptability												
Lethal	0.05	0.02	0.062	0.23	0.03	<0.001	0.04	0.01	<0.001	0.02	0.01	0.162
Nonlethal	0.002	0.03	0.926	0.17	0.04	<0.001	0.03	0.02	0.058	0.06	0.01	<0.001
Deer Concerns												
Direct	0.01	0.03	0.886	−0.03	0.04	0.426	0.01	0.02	0.485	0.01	0.01	0.251
Indirect	−0.01	0.02	0.780	0.07	0.03	0.016	−0.002	0.01	0.891	−0.001	0.01	0.904
Deer Attitudes	0.27	0.08	0.001	0.20	0.12	0.086	0.09	0.03	0.011	0.08	0.04	0.032
Hunting Beliefs	0.12	0.03	<0.001	0.33	0.04	<0.001	0.06	0.01	<0.001	0.07	0.02	<0.001
Political Ideology	−0.05	0.05	0.263	−0.10	0.06	0.086	−0.03	0.02	0.124	−0.04	0.02	0.110
Primary Self-Identity (Rural Resident)												
Farmer/Rancher	−0.77	0.28	0.005	0.02	0.37	0.954	−0.13	0.13	0.335	−0.30	0.12	0.013
Woodland Owner	0.14	0.21	0.489	−0.21	0.29	0.474	0.12	0.13	0.353	0.11	0.12	0.372
Deer Hunter	−0.21	0.28	0.443	−0.14	0.36	0.689	0.001	0.13	0.995	−0.01	0.09	0.882
Urban Resident	−0.27	0.24	0.271	−0.20	0.20	0.335	0.05	0.07	0.423	−0.01	0.09	0.939
Wildlife Value Orientation (Traditionalist)												
Mutualist	0.01	0.25	0.955	0.04	0.33	0.902	−0.04	0.12	0.728	0.10	0.12	0.389
Pluralist	0.19	0.16	0.230	−0.01	0.18	0.942	0.15	0.07	0.022	0.15	0.06	0.013
Distanced	−0.79	0.48	0.096	−0.37	0.45	0.417	−0.01	0.20	0.956	0.12	0.15	0.425
Organizational Membership (None)												
Hunting	−0.20	0.28	0.489	−0.16	0.38	0.678	−0.10	0.15	0.510	0.07	0.11	0.535
Environmental	0.17	0.27	0.529	0.23	0.36	0.533	0.14	0.11	0.226	0.25	0.11	0.020
Animal Welfare	0.17	0.26	0.510	−0.30	0.33	0.365	0.10	0.10	0.295	0.18	0.14	0.205
Allows Hunting (Yes)												
No	0.41	0.19	0.031	0.21	0.20	0.287	0.12	0.09	0.181	0.05	0.08	0.509
Cannot	0.70	0.19	<0.001	0.07	0.25	0.787	0.25	0.10	0.010	0.13	0.08	0.110
College Graduate (vs. No 4-yr Degree)												
	0.12	0.15	0.442	−0.14	0.15	0.374	0.20	0.06	0.001	0.16	0.06	0.006
Deer Hunter (vs. Not a Deer Hunter)												
	0.05	0.17	0.750	0.29	0.23	0.205	−0.21	0.09	0.022	−0.05	0.07	0.456
Woman (vs. Man)	−0.20	0.19	0.300	−0.03	0.21	0.903	0.05	0.07	0.488	−0.01	0.07	0.830
Constant	6.06	0.25	<0.001	0.87	0.30	0.004	0.11	0.12	0.347	0.53	0.11	<0.001

The respondent characteristics that influenced overall satisfaction were political ideology, wildlife value orientation, and allowing hunting on one’s property. A standard deviation change in respondent political ideology (1.5 units toward more conservative ideals) corresponded to a decrease in median predicted satisfaction by 0.44 units (*β* = −0.29; *p* = 0.024; Table 2). Respondents who held pluralist value orientations showed 1.17 units greater median predicted satisfaction with deer management than did those with traditionalist value orientations (*β* = 1.17; *p* = 0.002, Table 2). Compared to those who do allow hunting, respondents who could not allow hunting on their property due to unsuitable land or legal restrictions exhibited 1.29 units greater median satisfaction with deer management (*β* = 1.29, *p* = 0.009, Table 2).

### Predictors of Management Efficacy, Performance, and Trust (Satisfaction Components)

Among satisfaction index components, hunting beliefs were the only variable to significantly affect every component of the index. Increasingly positive beliefs about hunting increased median predicted ratings of management efficacy (*β* = 0.33, *p* < 0.001), the IN-DNR’s performance (*β* = 0.12, *p* < 0.001), agency trust (*β* = 0.06, *p* < 0.001), and informational trust (*β* = 0.07, *p* < 0.001; Table 3). Respondent levels of acceptability for deer management methods and concern about indirect impacts from deer were the only other variables to influence median management efficacy ratings. A standard deviation increase in the acceptability of lethal management methods (+3.4 units) and that of nonlethal management (+2.8 units) corresponded to 0.76-unit and 0.48-unit increases, respectively, in median management efficacy ratings (*β*_lethal_ = 0.23, *p* < 0.001; *β*_nonlethal_ = 0.17, *p* < 0.001; Table 3). Increasing levels of concern about indirect deer impacts by one standard deviation (+4.67 units) resulted in a 0.31-unit increase in the predicted median rating of management efficacy (*β* = 0.07; *p* = 0.016; Table 3). Neither respondent concerns about direct impacts from deer nor their general attitudes toward deer affected median management efficacy ratings (Table 3).

General attitudes toward deer affected ratings of the IN-DNR’s performance on deer management. A standard deviation change in attitudes toward deer (0.96 units more positive) corresponded to a 0.26-point higher median rating of the IN-DNR’s performance (*β* = 0.27, *p* = 0.001; Table 3). Performance ratings were also influenced by respondents’ self-identity and allowance of hunting on their property. Respondents identifying as farmers or ranchers provided the IN-DNR with significantly lower median performance ratings than did those identifying as rural residents (*β* = −0.77 *p* = 0.005; Table 3). Compared to respondents who do allow hunting on their property, respondents who do not allow hunting provided 0.41-point higher median ratings of the IN-DNR’s performance (*p* = 0.031) while those who could not allow hunting showed 0.70-point higher median performance ratings (*p* < 0.001; Table 3).

Examining trust components of the satisfaction index, increasing acceptability of lethal management methods by one standard deviation (+3.41 units) increased median agency trust by 0.14 units (*β* = 0.04; *p* < 0.001). A standard deviation increase in the acceptability of nonlethal management (+2.83 units) corresponded to a 0.16-unit increase in median levels of informational trust (*β* = 0.06; *p* < 0.001; Table 3). Increasingly positive attitudes toward deer by one standard deviation (+0.96 units) were associated with 0.08-unit increase in median agency trust (*β* = 0.09; *p* = 0.011) and a 0.07-unit increase in median informational trust (*β* = 0.08; *p* = 0.032; Table 3).

Consistent with their effects on the overall satisfaction index, respondent self-identity, allowance of hunting, and wildlife value orientation also influenced levels of trust in the IN-DNR and its information. Respondents who could not allow hunting on their property showed a 0.25-unit higher level of agency trust compared to those who do allow hunting (*p* = 0.010; Table 3). Farmers or ranchers placed significantly lower trust in the IN-DNR’s information than did rural residents (*β* = −0.30; *p* = 0.013; Table 3). Compared to traditionalist value orientations, pluralists showed higher median levels of trust in both the IN-DNR (*β* = 0.15, *p* = 0.022) and its information (*β* = 0.15, *p* = 0.013; Table 3).

Trust components of satisfaction were also affected by respondents’ organizational membership, college degree status, or identification as a deer hunter. Compared to those with no organizational membership, respondents belonging to a non-hunting environmental organization showed 0.25-units greater trust in information from the IN-DNR (*p* = 0.020; Table 3). College graduates were more trusting of the IN-DNR (*β* = 0.20, *p* = 0.001) and its information (*β* = 0.16, *p* = 0.006) than those without a 4-year degree (Table 3). Deer hunters, in contrast, were significantly less trusting of the IN-DNR than non-deer hunters (*β* = −0.21, *p* = 0.022; Table 3).

## Discussion

We formulated and analyzed a holistic measure of resident satisfaction with deer management. Satisfaction was strongly influenced by residents’ acceptability ratings of lethal and nonlethal management methods, their deer-related concerns, their underlying cognitions about deer or hunting, and their individual characteristics. Further, perceived efficacy of deer management methods, as a proxy for quality of the deer management service, was most significantly affected by residents’ acceptability levels and deer-related concerns. In contrast, IN-DNR performance, agency trust, and informational trust were influenced to a greater degree by attitudes toward deer, hunting beliefs, and resident characteristics. We discuss these effects in detail and their implications for understanding satisfaction with wildlife management more broadly.

Respondents’ acceptability of deer management methods significantly influenced their perceived efficacy of such methods, but in different ways. As respondents rated lethal methods increasingly acceptable, their perceived efficacy of all lethal methods also increased, whereas increasing acceptability of nonlethal methods mostly affected respondents’ perceived efficacy of alternative hunting programs like CHAPs (Online Resource 2). Normative beliefs, specifically those related to wildlife use or hunting, have consistently explained significant variation in the acceptability of lethal and nonlethal management approaches (Loker et al. 1999; Fulton et al. 2004; Whittaker et al. 2006; Bruskotter et al. 2009; Urbanek et al. 2015). Our analysis confirmed a consistently strong relationship between beliefs about hunting and perceived management efficacy, and such beliefs were moderately correlated with lethal (*r* = 0.23) and nonlethal (*r* = 0.28) management acceptability ratings. Intuitively, supportive beliefs about the effectiveness, humaneness, safety, and cultural importance of hunting—the items included in our composite variable—should lead to beliefs in the efficacy of lethal deer management approaches, as they typically involve some form of hunting. Alternatively, residents may be unaware of the efficacy of various management approaches, especially nonlethal methods (Kilpatrick et al. 2007). Underlying beliefs and attitudes about deer then become important cognitive factors influencing how residents appraise the efficacy and acceptability of unfamiliar management approaches (Whittaker et al. 2006, Fishbein and Ajzen 2010).

Satisfaction with deer management became more nuanced when considering landowners’ ability to allow hunting on their property. Psychological hazard-acceptance models posit that perceived control over wildlife management moderates the effect of trust in wildlife management agencies on an individual’s acceptance of wildlife-related risks or benefits (Zajac et al. 2012; Bruskotter and Wilson 2014). When an individual believes that they can act to manage wildlife and that their actions will produce desired outcomes, they place less importance on the actions of wildlife agencies and less trust in agency authority (Bruskotter and Wilson 2014). This aligns with our finding on the relationship between allowing licensed hunting on one’s property and satisfaction with the deer management agency. Respondents who cannot allow hunting—due to an unsuitable location or prohibition by local ordinances—consistently showed higher satisfaction, performance ratings, and trust in the IN-DNR compared to those who have control over that decision. This group also showed less concern about direct and indirect deer impacts than respondents who can and do control hunting on their land. Therefore, residents who have or perceive little control over deer hazards and benefits remain psychologically distanced from deer management at a local scale (Rotter 1966; Trope and Liberman 2010) and rely upon the managing agency more than do those who have control over their own lands.

People’s trust in wildlife management agencies typically correlates with their social identities and wildlife value orientations (Schroeder et al. 2021b). Our results confirm that residents who hunt deer, identify as a farmer or rancher, or orient to traditionalist values are generally less trusting of the agency and its information than other identities or value orientations. We also found that trust increases among residents with positive attitudes toward deer. Bruskotter et al. (2009) suggests that the effects of demographic characteristics on respondents’ acceptability of lethal wildlife management can become dampened by their attitudes toward and beliefs about a species, but this relationship has not been closely examined in either trust or satisfaction frameworks. With all four of these variables—self-identity, wildlife value orientation, attitudes toward deer, and hunting beliefs—included in our models, pluralist value orientations showed double the effect of attitudes toward deer on increasing trust in the management agency, and the farmer/rancher identity showed an effect three times larger than that of attitudes toward deer on informational trust but opposite in sign. Our findings suggest that even when considering cognitive variables (i.e., attitudes or beliefs), respondent values and identities retain a critical influence on their trust in wildlife agencies.

The relationship between trust in wildlife agencies and farmer/rancher or hunter identities warrants further examination. Previous studies have found that farmers’ trust in government erodes when farmers perceive the institutions in question to be physically or socially distant from them, even if those institutions actively support agricultural interests (Lubell 2007; Hall and Pretty 2008). Close personal contact with resource agency staff have predicted public trust and satisfaction with services, programs, or management strategies across social groups (Needham and Vaske 2008; Young et al. 2016). Social trust, built on these consistent interpersonal interactions, is more important than institutional trust for farmer participation in environmental programs, especially under high transaction costs like those associated with managing deer on private land (Mettepenningen et al. 2011; Selinske et al. 2015). Farmers and ranchers in our sample rated the IN-DNR’s deer management performance significantly lower than other self-identities and placed significantly less trust in its information. Such negative perceptions may result from frequent crop damage, frustration with deer impacts, and a lack of personal interactions with the IN-DNR (Stinchcomb et al. 2022). We know that interpersonal communication increases farmers’ trust in conservation-based agencies and their participation in decision-making processes (Breetz et al. 2005). Agency personnel also gain new knowledge from one-on-one conversations with landowners that translate to more effective conservation strategies (Kohl and Warner 2022). Thus, wildlife agency staff should make regular contact with farming and ranching constituents to gain their trust and improve deer management on private lands.

The low trust placed in wildlife management agencies by hunters may seem counterintuitive. Schroeder et al. (2021b) found hunters to have higher levels of trust in wildlife management agencies than livestock producers, stemming from hunters’ frequent interaction with agency staff and perceived value similarity with agency decisions. Even hunters who frequently interact with agency staff, however, can disagree with specific management strategies due to their high, and specific, expectations for deer management outcomes (Schroeder et al. 2017). Consequently, when hunters perceive a gap between their expectations and the condition of deer populations or hunting opportunities, they become less trusting of and less satisfied with management. In our study, significantly higher proportions of hunters were very concerned about hunting and perceived deer populations to be low compared to non-hunters, which suggests that hunters did not perceive their needs as being met by the IN-DNR. Not surprisingly, hunters were less trusting of the IN-DNR and less satisfied with deer management than the non-hunting public.

The above evidence suggests that regular interpersonal interactions between wildlife agencies and members of the public is critical. Increasing contact, communication, and transparency will help to increase residents’ perceived control over management (Slagle et al. 2013), participation in public decision-making processes (Willcox and Giuliano 2014), perceived fairness of management (Young et al. 2016), and overall trust in management agencies (Manfredo et al. 2017; Schmidt et al. 2018). We also know that knowledge exchange and power-sharing contribute to long-term trust (Denize and Young 2007; Young et al. 2016), and this is best served through interpersonal contact. But contact with every individual can be expensive and impractical for state agencies to implement at a large scale (Caudell and Vaught 2019). Engaging with key leaders of organizations, social groups, or communities can build capacity and impetus for local wildlife management (Raik et al. 2008), facilitate the diffusion of management techniques and information within communities (Valente and Davis 1999; van Eeden et al. 2021), and shift control over management to local groups, which may enhance public trust in government agencies (Redpath et al., 2017; Bogezi et al. 2021). In the absence of direct contact with individuals, agency staff should form relationships with relevant social leaders. Leaders can represent physical communities, stakeholder groups, or other organizations, but should ideally have a working and trusting relationship with target individuals, like that between County Extension agents and farmers or ranchers (Bogezi et al., 2021). Both the cost-effectiveness of this strategy and how agencies are shifting their internal and public-facing cultures, values, and services require further evaluation.

We recommend caution when interpreting our results to broader populations, particularly in urban contexts. Compared to census data for Indiana, our sample contained significantly greater proportions of white/Caucasian, male, well-educated, high-income, and rural-dwelling residents (Table 1). These differences are directly attributable to our sampling strategy, which intentionally targeted rural forestland and agricultural properties, as well as existing constituents (i.e., license-holders) of the Indiana DFW. We encourage future research to focus on non-traditional wildlife constituents in urban areas and compare their perceptions, needs, and concerns with those from more traditional constituents in rural areas. Furthermore, we encourage wildlife management agencies to engage with diverse groups beyond their traditional stakeholders and to clearly define satisfaction with wildlife management to truly embrace the Public Trust Doctrine (Jacobson et al. 2010; Coleman et al. 2019). Although Decker and Chase (1997) voiced their concerns about “the spread of a ‘customer service and satisfaction’ attitude among wildlife agencies” (p. 794) 25 years ago, many agencies continue to operate using a market-based model of public administration, which treats the public as “customers” (Kelly 2005; Funck and Karlsson 2020). However, public agencies are not private sector businesses and the purpose of wildlife management is not to sell hunting licenses nor issue wildlife depredation permits. These customer-oriented practices can be means to an end—e.g., managing natural resources in the public trust—but not the ends themselves. When wildlife agencies try to increase public satisfaction with deer management, we advise them to remember that “deer are public trust resources, wildlife agency leaders are the trustees, professional wildlife managers are the trust managers, and *[all] citizens* are the beneficiaries of the trust” (quote from an anonymous reviewer, emphasis added).

Although we planned to analyze trust in deer management and perceptions of agency performance, our interest in multidimensional satisfaction largely arose after our survey was developed. We thus did not measure components of satisfaction a priori. Future research should develop and test indices prior to implementation in a study population (Hinkin 1995; Clark and Watson 1995; Reise et al. 2000). Furthermore, while moderate to high correlations are preferred to ensure variables composing an index are measuring the same or similar constructs (Clark and Watson 1995), our data showed relatively low correlations between management efficacy and the remaining index variables (*r* = 0.21–0.25). Performance or service quality and agency or informational trust may be different constructs influenced by different variables. Whereas normative judgements and concerns about deer—related to cognitive accessibility, perceived control, and psychological distance—affected performance and quality measures, demographic characteristics exerted the strongest influence on trust. Therefore, future research may need to treat these two components of satisfaction as distinct concepts but synthesize them together.

Even if performance and trust represent separate constructs, it remains important for wildlife and resource management agencies to quantify the determinants of public satisfaction with their services and governance (Gutek et al. 1983). Examining variation in the what and the why of public (dis)satisfaction is crucial to improving wildlife and resource management services (Kelly 2005; Selinske et al. 2015; Coleman et al. 2019). But survey questions about satisfaction with wildlife management continue to use unidimensional scales like a rating of one to ten. Replacing or supplementing these performance scores with a series of questions that capture multiple dimensions of satisfaction with and trust in wildlife agencies will help agencies better explain the variation in subjective ratings among survey respondents and manage (dis)satisfaction across a wider public. As Pomeranz et al. (2021) recently noted, good governance based in public trust thinking constitutes a continual practice for wildlife agencies, rather than a clear objective to be achieved. Expanding agency conceptions of public trust and satisfaction will help to expand the market-based model that agencies consider in their administration of wildlife resources. These expansions represent an important step toward practicing a more inclusive, beneficiary model of wildlife governance (Decker et al. 2019).

## Supplementary Information

Supplementary Material
