# Supplementary material for: Expanding and Evaluating Public Satisfaction with Wildlife Governance: Insights from Deer Management in Indiana, USA
Source: Environ Manage. 2022 Aug 23;70(5):780–92. doi: 10.1007/s00267-022-01698-5 (PMC9519643; doi:10.1007/s00267-022-01698-5)
Supplement: Supplementary file 1 — Supplementary Material [file 267_2022_1698_MOESM1_ESM.docx]

**Supplementary Material**

**Expanding and evaluating public satisfaction with wildlife governance: insights from deer management in Indiana**

Taylor R. Stinchcomb^1^, Zhao Ma, Robert K. Swihart, Joe N. Caudell

^1^Corresponding author. Affiliation: Purdue University, Department of Forestry & Natural Resources. Email: [tstinchcomb@pm.me](mailto:tstinchcomb@pm.me)

*Online Resource 1. Descriptive Statistics on Model Variables*

Across our final sample, respondents showed positive satisfaction with deer management ($\bar{x}$ = 9.2, sd = 5.5, Table S1). Most respondents found existing deer management methods to be very effective, effective, or neither effective nor ineffective (Table S1). The average rating given to the IN-DNR for deer management was a 6.7 (sd = 2.1). Most respondents trusted the technical competency of the IN-DNR but reported slightly lower trust in the agency’s procedural fairness and value similarity (Table S1). About a third of respondents found IN-DNR sources of deer information to be somewhat trustworthy and another third found these sources to be very trustworthy (Table S1). On our independent variables, respondents found lethal management methods to be slightly acceptable on average ($\bar{x}$ = 0.24, sd = 3.4) and nonlethal methods to be unacceptable on average ($\bar{x}$ = -1.01, sd = 2.8, Table S2). Respondents held slightly positive attitudes towards deer ($\bar{x}$ = 0.71, sd = 0.96) and generally agreed with positive beliefs about hunting ($\bar{x}$ = 4.43, sd = 3.36 Table S2). Respondents were concerned (25%) or very concerned (32%) about deer-vehicle collisions but split in their concern about damage to crops, gardens, or landscaping (Table S2). Respondents were neutral or concerned about most indirect deer-related impacts except for hunting opportunities, about which a third were not concerned (Table S2).

Table S1. Description, summary statistics, and factor loadings for variables comprising the satisfaction index and independent variables of interest.

| **Variable** | **Description of variable and value levels (%of responses at each level)** | **Mean** | | **S.D.** | **Factor loading** |
| --- | --- | --- | --- | --- | --- |
|  |  |  |  | |  |
| **Overall Satisfaction** | Index comprised of the variables below. Final values range from -10.33 to 21.33. | 9.16 | 5.47 | | n/a |
| Management Efficacy | Composite variable of the items for *"how ineffective or effective do you consider existing actions to manage deer in Indiana?"* | 2.35 | 2.91 | | n/a |
| Licensed Hunting | -2 = Very ineffective (2%), -1 (3%); 0 = neither (15%); 1 (31%); 2 = Very effective (45%) | 1.18 | 0.96 | | 0.64 |
| Culling deer in specific areas | -2 = Very ineffective (5%), -1 (6%); 0 = neither (30%); 1 (29%); 2 = Very effective (27%) | 0.69 | 1.09 | | 0.76 |
| Urban Deer Reduction Zones (DRZs) | -2 = Very ineffective (5%), -1 (7%); 0 = neither (35%); 1 (25%); 2 = Very effective (23%) | 0.58 | 1.08 | | 0.83 |
| Community Hunting Access Programs (CHAPs) | -2 = Very ineffective (7%), -1 (9%); 0 = neither (41%); 1 (20%); 2 = Very effective (18%) | 0.35 | 1.10 | | 0.80 |
| Provide Advice or Information | -2 = Very ineffective (5%), -1 (9%); 0 = neither (35%); 1 (24%); 2 = Very effective (22%) | 0.51 | 1.12 | | 0.64 |
| IN-DNR rating | *On a scale of 1 to 10, how well is the IN-DNR managing deer in Indiana?* | 6.67 | 2.11 | | n/a |
| Agency Trust | Composite variable of the items for *"How much do you distrust or trust the Indiana DNR to do the following?"* | 0.51 | 0.94 | | n/a |
| Employ people with the scientific expertise necessary to manage deer in Indiana | -2 = Distrust (4%), -1 (6%); 0 = neither (28%); 1 (31%); 2 = Trust (26%) | 0.74 | 1.05 | | 0.83 |
| Employ people who know what needs to be done to manage deer in Indiana | -2 = Distrust (4%), -1 (8%); 0 = neither (26%); 1 (33%); 2 = Trust (24%) | 0.69 | 1.06 | | 0.87 |
| Listen to the public | -2 = Distrust (8%), -1 (11%); 0 = neither (33%); 1 (29%); 2 = Trust (15%) | 0.35 | 1.12 | | 0.87 |
| Treat members of the public equally | -2 = Distrust (7%), -1 (10%); 0 = neither (31%); 1 (28%); 2 = Trust (19%) | 0.46 | 1.14 | | 0.84 |
| Communicate unbiased information to the public | -2 = Distrust (5%), -1 (9%); 0 = neither (31%); 1 (30%); 2 = Trust (21%) | 0.54 | 1.10 | | 0.89 |
| Share similar values as me for deer management | -2 = Distrust (6%), -1 (9%); 0 = neither (36%); 1 (28%); 2 = Trust (17%) | 0.45 | 1.07 | | 0.86 |
| Consider the opinions and needs of people like me when making deer management decisions | -2 = Distrust (7%), -1 (12%); 0 = neither (34%); 1 (27%); 2 = Trust (17%) | 0.38 | 1.12 | | 0.87 |
| Informational Trust | Composite variable of the items for *"How trustworthy do you consider the following sources of deer management information in Indiana?"* | 0.89 | 0.88 | | n/a |
| IN-DNR website or newsletters | -2 = Very untrustworthy (2%), -1 (4%); 0 = neither (28%); 1 (33%); 2 = Very trustworthy (27%) | 0.84 | 0.96 | | 0.89 |
| District Conservation Officers | -2 = Very untrustworthy (2%), -1 (4%); 0 = neither (23%); 1 (34%); 2 = Very trustworthy (30%) | 0.92 | 0.97 | | 0.90 |
| District Wildlife Biologists | -2 = Very untrustworthy (2%), -1 (3%); 0 = neither (25%); 1 (33%); 2 = Very trustworthy (30%) | 0.92 | 0.96 | | 0.93 |
| District Foresters | -2 = Very untrustworthy (2%), -1 (4%); 0 = neither (27%); 1 (32%); 2 = Very trustworthy (28%) | 0.86 | 0.98 | | 0.91 |

Table S2. Description, summary statistics, and factor loadings for independent variables of interest in quantile regression models.

| **Independent Variable** | **Description of variable and value levels (%of responses at each level)** | **Mean** | | **S.D.** | **Factor loading** |
| --- | --- | --- | --- | --- | --- |
| **Management Acceptability** | *In your area, how unacceptable or acceptable do you consider the following potential actions to manage deer?* |  |  | |  |
| Lethal | Composite variable of lethal methods. | 0.24 | 3.41 | | n/a |
| Increasing licensed hunting | -2 = very unacceptable (14%); -1 (10%); 0 = neither (23%); 1 (23%); 2 = very acceptable (26%) | 0.38 | 1.37 | | 0.89 |
| Culling deer populations | -2 = very unacceptable (19%); -1 (15%); 0 = neither (28%); 1 (19%); 2 = very acceptable (15%) | -0.05 | 1.34 | | 0.85 |
| Creating a Community Hunting Access Program | -2 = very unacceptable (18%); -1 (15%); 0 = neither (33%); 1 (17%); 2 = very acceptable (13%) | -0.09 | 1.28 | | 0.82 |
| Nonlethal | Composite variable of nonlethal methods. | -1.01 | 2.83 | | n/a |
| Using contraception to control deer | -2 = very unacceptable (43%); -1 (15%); 0 = neither (22%); 1 (9%); 2 = very acceptable (8%) | -0.80 | 1.31 | | 0.79 |
| Trapping & relocating deer | -2 = very unacceptable (38%); -1 (18%); 0 = neither (22%); 1 (11%); 2 = very acceptable (8%) | -0.69 | 1.32 | | 0.86 |
| Providing advice or information about deer | -2 = very unacceptable (8%); -1 (9%); 0 = neither (33%); 1 (24%); 2 = very acceptable (23%) | 0.47 | 1.18 | | 0.51 |
| **Deer Concerns** | *How concerned are you about the following issues related to deer?* |  |  | |  |
| Direct | Composite variable of direct concerns | 0.33 | 3.08 | | n/a |
| Vehicle collisions with deer | -2 = Not at all concerned (7%), -1 (10%); 0 = neutral (24%); 1 (25%); 2 = Very concerned (32%) | 0.66 | 1.23 | | 0.76 |
| Deer damage to crops, gardens, or landscaping | -2 = Not at all concerned (17%), -1 (18%); 0 = neutral (28%); 1 (17%); 2 = Very concerned (18%) | 0.04 | 1.33 | | 0.82 |
| Deer damage to forests | -2 = Not at all concerned (26%), -1 (19%); 0 = neutral (28%); 1 (15%); 2 = Very concerned (10%) | -0.37 | 1.29 | | 0.74 |
| Indirect | Composite variable of indirect concerns | 1.54 | 4.67 | | n/a |
| Disease transmission related to deer | -2 = Not at all concerned (11%), -1 (12%); 0 = neutral (22%); 1 (26%); 2 = Very concerned (27%) | 0.47 | 1.32 | | 0.63 |
| Current deer population sizes | -2 = Not at all concerned (10%), -1 (11%); 0 = neutral (29%); 1 (25%); 2 = Very concerned (23%) | 0.40 | 1.25 | | 0.64 |
| Deer hunting opportunities | -2 = Not at all concerned (31%), -1 (11%); 0 = neutral (17%); 1 (16%); 2 = Very concerned (23%) | -0.14 | 1.57 | | 0.76 |
| Deer welfare | -2 = Not at all concerned (6%), -1 (8%); 0 = neutral (26%); 1 (29%); 2 = Very concerned (28%) | 0.67 | 1.16 | | 0.77 |
| Managing deer in urban areas | -2 = Not at all concerned (14%), -1 (14%); 0 = neutral (32%); 1 (22%); 2 = Very concerned (17%) | 0.15 | 1.27 | | 0.50 |
| **Deer Attitudes** | Composite variable of the items for *"In general, do you think of deer as:"* | 0.71 | 0.96 | | n/a |
| Bad/Good | -2 = Very Bad (1%); -1 = slightly bad (4%); 0 = neither (28%); 1 = slightly good (20%); 2 = very good (42%) | 1.02 | 1.02 | | 0.81 |
| Dangerous/Harmless | -2 = Very dangerous (4%); -1 = slightly dangerous (11%); 0 = neither (28%); 1 = slightly harmless (23%); 2 = very harmless (30%) | 0.67 | 1.14 | | 0.81 |
| Detrimental/Beneficial | -2 = Very detrimental (4%); -1 = slightly detrimental (11%); 0 = neither (32%); 1 = slightly beneficial (22%); 2 = very beneficial (26%) | 0.58 | 1.12 | | 0.89 |
| Nuisance/Asset | -2 = Very nuisance (6%); -1 = slightly nuisance (14%); 0 = neither (27%); 1 = slightly asset (21%); 2 = very asset (28%) | 0.54 | 1.22 | | 0.88 |
| **Independent Variable** | **Description of variable and value levels (%of responses at each level)** | **Mean** | | **S.D.** | **Factor loading** |
| **Hunting Beliefs** | Composite variable of the items for *"To what extent do you agree with the following statements about deer hunting?"* | 4.43 | 3.36 | | n/a |
| Hunting is the most effective way to control deer populations | -2 = Strongly disagree (3%); -1 (4%); 0 = neither (16%); 1 (29%); 2 = strongly agree (46%) | 1.13 | 1.04 | | 0.82 |
| Hunters will harvest enough deer to control population numbers | -2 = Strongly disagree (4%); -1 (9%); 0 = neither (28%); 1 (29%); 2 = strongly agree (27%) | 0.69 | 1.10 | | 0.64 |
| Most hunters are responsible and safe with their weapons | -2 = Strongly disagree (3%); -1 (7%); 0 = neither (20%); 1 (34%); 2 = strongly agree (33%) | 0.90 | 1.05 | | 0.73 |
| Hunting is a humane way to control deer populations | -2 = Strongly disagree (3%); -1 (4%); 0 = neither (15%); 1 (28%); 2 = strongly agree (48%) | 1.16 | 1.04 | | 0.87 |
| Hunting does not present a safety risk to myself or others* | -2 = Strongly disagree (6%); -1 (12%); 0 = neither (20%); 1 (25%); 2 = strongly agree (35%) | 0.72 | 1.24 | | 0.52 |
| Hunting is an important part of Indiana's culture | -2 = Strongly disagree (2%); -1 (3%); 0 = neither (13%); 1 (25%); 2 = strongly agree (54%) | 1.29 | 0.97 | | 0.77 |

**Reverse-coded to align with the direction of other items. Original wording: “Hunting presents a safety risk to myself or others.”*

*Online Resource 2. Item-level analysis of the management efficacy variable*

**Methods**

Due to differing factor loadings for items comprising management efficacy, we conducted an item-level analyses of this variable using the same predictors used in quantile regression models for overall satisfaction and the components of satisfaction. Since survey items were Likert-type scales, we conducted multinomial logistic regressions on each item via the *mlogit* command in STATA after finding that multiple predictor variables violated the proportional odds assumption for ordinal models (Long and Freese 2006). We then examined the marginal effects of independent variables on the predicted probabilities of rating each management method as ineffective, effective, or neither. We collapsed the two highest (“very effective,” “effective”) categories into one “effective” rating and the two lowest categories (“very ineffective,” “ineffective”) into one “ineffective” rating to reduce the complexity of analysis. We present the marginal effect of a standard deviation change in significant predictor variables, as this allows us to compare the magnitude of each variable’s effect on predicted probabilities of rating management methods as ineffective, neither, or effective across models (Mize et al. 2019).

**Results**

When examining item-level effects, lethal and nonlethal acceptability ratings showed different relationships with the perceived efficacy of various management methods. Greater acceptance of lethal management methods was associated with a greater predicted probability of rating all lethal management approaches as effective, and lower probabilities of rating them neither or ineffective (Table S2). By comparison, greater acceptance of nonlethal management decreased the probability of ineffective ratings for all alternative deer management approaches (i.e., Deer Reduction Zones (DRZs), CHAPs, and information provision) and increased the probability of effective ratings for CHAPs and information provision (Table S2).

As respondents’ level of concern about indirect deer impacts increased, residents were more likely to rate hunting and culling methods as ineffective (p_hunt_ = 0.02, p_cull_ = 0.003; Table S2). They were also more likely than residents with lower levels of indirect deer concerns to rate alternative lethal methods of DRZs and CHAPs as effective (p_DRZ_ = 0.01, p_CHAP_ < 0.001; Table S2). In the case of DRZs, however, increasing indirect deer concerns also increased the probability of an ineffective rating (Table S2).

Respondent beliefs about hunting showed significant effects on the efficacy ratings of all management methods. Greater agreement with beliefs about hunting corresponded to reduced probability of rating lethal methods as ineffective (all slopes -0.02 or -0.03, p < 0.001 to 0.03) or as neither effective nor ineffective by 0.07 to 0.1 (slopes from -0.07 to -0.10, all p < 0.001), and increased probability of rating them as effective (slopes from 0.10 to 0.13, all p < 0.001; Table S2). Increasingly positive beliefs about hunting also increased the probability of an effective rating for information provision (b = 0.09, p < 0.001; Table S2).

Respondent self-identities mostly affected efficacy ratings for alternative hunting programs like DRZs and CHAPs. Farmers/ranchers and urban residents, separately, were more likely than, deer hunters (p_farm_ = 0.01, p_urb_ = 0.02), and rural residents (p_farm_ = 0.013, p_urb_ = 0.02) to rate DRZs as ineffective (Table S2). Farmers/ranchers were also more likely than woodland owners to rate DRZs as ineffective (p = 0.04) while urban residents were more likely than rural residents to rate CHAPs as ineffective (p= 0.05, Table S2).

Although allowing hunting on one’s property did not significantly affect the composite management efficacy variable, at the item-level, residents who could not allow hunting were significantly less likely to rate licensed hunting as ineffective than those who allowed hunting (b = -0.04, p = 0.01) and those who did not allow hunting (b = -0.04, p = 0.02; Table S2).

|  | **Licensed Hunting** | | | **Culling** | | | **DRZ** | | | **CHAP** | | | **Providing Information** | | |
| --- | --- | --- | --- | --- | --- | --- | --- | --- | --- | --- | --- | --- | --- | --- | --- |
| **Predictor Variable** | Ineffective | Neither | Effective | Ineffective | Neither | Effective | Ineffective | Neither | Effective | Ineffective | Neither | Effective | Ineffective | Neither | Effective |
| Acceptability, Lethal Control |  | -0.02* | 0.03** | -0.04** | -0.06** | 0.10** | -0.03** | -0.05** | 0.08** | -0.06** | -0.07** | 0.12** |  |  |  |
| Acceptability, Nonlethal Control |  |  |  | -0.03** |  |  | -0.03** | 0.04* |  | -0.05** |  | 0.04* | -0.06** | -0.06** | 0.12** |
| Direct deer concerns |  |  |  | -0.02* |  |  | -0.03* |  |  |  |  | -0.04* |  |  |  |
| Indirect deer concerns | 0.03* | -0.03* |  | 0.04* | -0.05* |  | 0.03* | -0.07** | 0.04* |  | -0.06** | 0.07** | -0.03* | -0.05** | 0.08** |
| General deer attitudes | -0.01* | -0.02* | 0.04** |  |  |  |  |  |  |  |  |  |  | -0.03* | 0.05** |
| Hunting beliefs | -0.03** | -0.07** | 0.10** | -0.03** | -0.09** | 0.11** | -0.02* | -0.10** | 0.12** | -0.03* | -0.10** | 0.13** |  | -0.08** | 0.09** |
| College Graduate |  |  | 0.04* |  |  | 0.06* |  |  |  |  | 0.06* |  |  |  |  |
| Woman |  | -0.04* |  |  |  |  | 0.05* |  |  |  |  |  |  |  |  |
| *Allows Hunting* |  |  |  |  |  |  |  |  |  |  |  |  |  |  |  |
| Cannot vs. Yes | -0.04* |  |  |  |  |  |  |  |  | -0.06* |  |  |  |  |  |
| Cannot vs. No | -0.04* |  |  |  |  |  |  |  |  |  |  |  |  |  |  |
| *Organizational Membership* |  |  |  |  |  |  |  |  |  |  |  |  |  |  |  |
| Hunting vs. None |  | -0.09* | 0.10* |  | -0.09* | 0.10* |  |  |  |  |  |  |  |  |  |
| Environmental vs. Hunting |  |  |  |  |  |  |  |  |  |  |  |  |  |  |  |
| *Respondent Self-Identity^a^* |  |  |  |  |  |  |  |  |  |  |  |  |  |  |  |
| WLO vs. Farmer/Rancher |  |  |  |  |  |  | -0.08* |  |  |  |  |  |  |  |  |
| Deer Hunter vs. Farmer/Rancher |  |  |  |  |  |  | -0.10* |  |  |  |  |  |  |  |  |
| Deer Hunter vs. WLO |  |  |  |  |  | -0.12* |  |  |  |  |  |  |  |  |  |
| Rural Resident vs. Farmer/Rancher |  |  |  |  |  |  | -0.08* |  |  |  |  |  |  |  |  |
| Urban Resident vs. Deer Hunter |  |  |  |  |  |  | 0.08* |  |  |  |  |  |  |  |  |
| Rural vs. Urban Resident |  |  |  |  |  |  | -0.07* |  |  | -0.056* |  |  |  |  |  |
| *Value Orientation* |  |  |  |  |  |  |  |  |  |  |  |  |  |  |  |
| Pluralist vs. Traditionalist |  |  |  |  |  |  |  |  |  |  | 0.66* | -0.061* |  |  |  |
| Distanced vs. Traditionalist |  |  |  |  |  |  |  |  |  |  |  |  |  | 0.147* |  |
| Distanced vs. Mutualist |  |  |  |  |  |  |  |  |  |  |  |  |  | 0.163* | -0.136* |

Table S2. Marginal effects of a standard deviation change in significant predictor variables on predicted probabilities of management efficacy ratings. Only significant results are presented: * p < 0.05; ** p < 0.001.

^a^Second identity listed is the base category to which marginal effect should be compared. A negative sign indicates less likely than the base category, while a positive sign indicates more likely than the base category to choose the corresponding rating. E.g., WLO = 0.08 times less likely than farmer/ranchers or urban resident = 0.08 times more likely than deer hunter to rate DRZs as ineffective. WLO = Woodland Owner.

**References**

Long, J.S., and J. Freese. 2006. *Regression Models for Categorical Dependent Variables
Using Stata*. 2nd ed. College Station, TX: Stata Press.

Mize, T. D., L. Doan, and J. S. Long. 2019. A general framework for comparing predictions and marginal effects across models. *Sociological Methodology* 49:152–189.
